# Differential Effects of Insulin-Deficient Diabetes Mellitus on Visceral vs. Subcutaneous Adipose Tissue—Multi-omics Insights From the Munich MIDY Pig Model

**DOI:** 10.3389/fmed.2021.751277

**Published:** 2021-11-23

**Authors:** Florian Flenkenthaler, Erik Ländström, Bachuki Shashikadze, Mattias Backman, Andreas Blutke, Julia Philippou-Massier, Simone Renner, Martin Hrabe de Angelis, Rüdiger Wanke, Helmut Blum, Georg J. Arnold, Eckhard Wolf, Thomas Fröhlich

**Affiliations:** ^1^Laboratory for Functional Genome Analysis (LAFUGA), Gene Center, Ludwig-Maximilians-Universität (LMU) Munich, Munich, Germany; ^2^German Center for Diabetes Research (DZD), Oberschleißheim, Germany; ^3^Gene Center, Graduate School of Quantitative Biosciences Munich (QBM), Ludwig-Maximilians-Universität (LMU) Munich, Munich, Germany; ^4^Helmholtz Zentrum München, Institute of Experimental Genetics, Oberschleißheim, Germany; ^5^Department of Veterinary Sciences, Gene Center, Institute for Molecular Animal Breeding and Biotechnology, Ludwig-Maximilians-Universität (LMU) Munich, Munich, Germany; ^6^Center for Innovative Medical Models (CiMM), Ludwig-Maximilians-Universität (LMU) Munich, Oberschleißheim, Germany; ^7^Helmholtz Zentrum München, Institute of Experimental Genetics, Technical University of Munich, Munich, Germany; ^8^Center for Clinical Veterinary Medicine, Institute of Veterinary Pathology, Ludwig-Maximilians-Universität (LMU) Munich, Munich, Germany

**Keywords:** diabetes, insulin deficiency, adipose tissue, proteome, transcriptome, pig model, biobank

## Abstract

Adipose tissue (AT) is no longer considered to be responsible for energy storage only but is now recognized as a major endocrine organ that is distributed across different parts of the body and is actively involved in regulatory processes controlling energy homeostasis. Moreover, AT plays a crucial role in the development of metabolic disease such as diabetes. Recent evidence has shown that adipokines have the ability to regulate blood glucose levels and improve metabolic homeostasis. While AT has been studied extensively in the context of type 2 diabetes, less is known about how different AT types are affected by absolute insulin deficiency in type 1 or permanent neonatal diabetes mellitus. Here, we analyzed visceral and subcutaneous AT in a diabetic, insulin-deficient pig model (MIDY) and wild-type (WT) littermate controls by RNA sequencing and quantitative proteomics. Multi-omics analysis indicates a depot-specific dysregulation of crucial metabolic pathways in MIDY AT samples. We identified key proteins involved in glucose uptake and downstream signaling, lipogenesis, lipolysis and β-oxidation to be differentially regulated between visceral and subcutaneous AT in response to insulin deficiency. Proteins related to glycogenolysis, pyruvate metabolism, TCA cycle and lipogenesis were increased in subcutaneous AT, whereas β-oxidation-related proteins were increased in visceral AT from MIDY pigs, pointing at a regionally different metabolic adaptation to master energy stress arising from diminished glucose utilization in MIDY AT. Chronic, absolute insulin deficiency and hyperglycemia revealed fat depot-specific signatures using multi-omics analysis. The generated datasets are a valuable resource for further comparative and translational studies in clinical diabetes research.

## Introduction

Adipose tissue is a major player in whole body energy homeostasis and regulation of metabolic functions. It serves as storage of surplus energy in the form of triglycerides in adipocytes and controls lipid mobilization during fasting by releasing free fatty acids (1, 2). With the discovery of adipocyte-derived factors such as leptin, adiponectin, and resistin, adipose tissue is increasingly recognized as a complex endocrine organ. Via adipokine signaling, adipose tissue is able to communicate with many organs like the liver, pancreas, muscle, and brain, and is therefore able to modulate systemic metabolism (3–6). Thus, adipose tissue dysfunction plays an important role in the pathogenesis of metabolic disorders, such as obesity, cardiovascular disease, insulin resistance, and diabetes mellitus (7–9). How adipose tissue specifically contributes to the pathogenesis of metabolic diseases is however highly complex and varies between different fat depots (10–12). It is thought that visceral adipose tissue is more likely to contribute to the pathogenesis of insulin resistance and type 2 diabetes mellitus (13, 14), while accumulation of subcutaneous fat has even been reported to reduce metabolic disease risk (15–17).

Several recent studies have analyzed adipose tissue proteomes in the context of type 2 diabetes mellitus to get a more global understanding of adipose tissue (dys-)function in states of insulin resistance and hyperinsulinemia (18–22). However, molecular consequences of chronic insulin deficiency on adipose tissue depots remain poorly explored.

As a large animal model for mutant *INS* gene induced diabetes of youth (MIDY), we generated *INS*^C94Y^ transgenic pigs, which are characterized by reduced body weight and β-cell mass, impaired insulin secretion with resulting hypoinsulinemia, and elevated blood glucose levels (23). Moreover, the model develops diabetes-associated alterations in heart (24), retina (25), immune cells (26), and liver (27). For studying long-term consequences of severe insulin-deficient diabetes mellitus (SIDD) (28), we established a biobank of 2-year-old MIDY pigs and healthy littermate controls following the principles of random systematic sampling (29).

In the current study, we performed label-free quantitative proteomics and RNA-sequencing of mesenteric visceral adipose tissue (MAT) and abdominal subcutaneous adipose tissue (SCAT). Using this holistic multi-omics approach, we report marked adipose tissue depot-specific responses to insulin deficiency and chronic hyperglycemia, and provide new insights into the molecular pathology of insulin-deficient diabetes in adipose tissue depots.

## Materials and Methods

### Biological Samples

Adipose tissue samples were obtained from the Munich MIDY Pig Biobank (29). Mesenteric visceral adipose tissue (MAT) and abdominal subcutaneous adipose tissue (SCAT) samples were collected from 2-year-old female MIDY pigs (*n* = 4) and female WT littermates (*n* = 5) by systematic random sampling (30). Tissue specimen were shock-frozen and stored at −80°C. To minimize variations induced by sample collection, two samples collected from the same animal but from different areas, were pooled separately for MAT and SCAT prior to Omics analysis.

### Proteomics

Frozen tissue samples were homogenized in 1% sodium deoxycholate (SDC) and 50 mM ammonium bicarbonate (ABC) using an ART-Miccra D-8 homogenizer (ART Prozess- & Labortechnik) at a speed of 23,500 rpm for two cycles of 1 min. Samples were kept on ice for 30 min and centrifuged at 16,000 × *g* for 5 min. The aqueous phase beneath the top lipid layer was carefully taken and transferred to a new test tube. Protein concentrations were determined using a NanoDrop ND-1000 spectrophotometer (Marshall Scientific) at 280 nm. Fifty microgram of protein was reduced with 4 mM dithiothreitol (DTT) and 2 mM tris(2-carboxyethyl)phosphine (TCEP) at 56°C for 30 min and alkylated with 8 mM iodoacetamide (IAA) at room temperature in the dark. DTT was added to a final concentration of 10 mM to quench residual IAA during 15 min incubation in the dark. Proteins were digested with 1 μg LysC (Wako) for 4 h followed by digestion with 1 μg modified porcine trypsin (Promega) for 16 h at 37°C. SDC was removed by acid precipitation as described elsewhere (31, 32).

Nano-liquid chromatography–tandem mass spectrometry (LC–MS/MS) analysis was performed on an Q Exactive HF-X mass spectrometer equipped with an UltiMate 3000 nano LC system (Thermo Scientific) as previously described (33). Briefly, 1.5 μg of peptides were separated on a 50 cm column (PepMap RSLC C18, 75 μm ID, 2 μm; Thermo Scientific) using linear gradients from 5 to 25% solvent B (0.1% formic acid in acetonitrile) in 160 min and from 25 to 40% solvent B in 10 min with a flow rate of 250 nl min^−1^. Spectra were acquired in data-dependent mode in cycles of one full scan in the range of 300–1,600 *m/z* at a resolution of 60,000, followed by MS/MS scans of the 15 most intense peaks at a resolution of 15,000.

Raw MS data were processed with MaxQuant (v. 1.6.7.0), using the integrated Andromeda search engine (34) and the NCBI RefSeq Sus scrofa database (v. 2020-11-12). Identifications were filtered to 1% false discovery rate (FDR) at peptide and protein level. Statistics and data visualization were performed in R (35). MS-EmpiRe was used to detect differentially abundant proteins (36). Reverse peptides, contaminants and identifications only by site were excluded for quantification. Proteins were quantified with at least two peptides with a minimum of two replicate measurements in each condition. For peptides with measurements in all replicates of one condition and insufficient measurements in the other condition, missing values were imputed from normal distribution (shift = 1.8, scale = 0.3) using the DEP package (37). Proteins were considered as significantly changed in abundance with a Benjamini-Hochberg-adjusted *P*-value < 0.05 and a fold change above 1.3.

### Transcriptomics

After weighing the frozen tissue, corresponding volumes of ice-cold Trizol reagent (Invitrogen Life Technologies) were added (1 ml Trizol reagent per 100 mg tissue). Tissue was immediately homogenized using the Heidolph Silents Crusher M. RNA of homogenized tissue was isolated using the Maxwell RSC miRNA Tissue Kit (Promega). Manufacturer's instructions were followed except for the following changes: the homogenized tissue was in Trizol to which corresponding volumes of 1-thioglycerol were added. Isolated RNA was additionally digested with DNase I (Thermo Scientific) and purified using Agencourt RNAClean XP beads (Beckman Coulter) following manufacturer's instructions. Subsequently, the RNA was quantified (Nanodrop) and quality controlled on an RNA 6000 Nano Chip using a Bioanalyzer (Agilent). Finally, 120 ng high quality total RNA (RIN > 8.0) was used for generating sequencing libraries using the Sense mRNA Seq Library Prep Kit V2 for Illumina platforms (Lexogen) following manufacturer's instructions. Libraries were quantified and quality controlled on the Bioanalyzer (Agilent) and finally sequenced on an Illumina HiSeq1500 machine (single end read, 100 nt).

After demultiplexing obtained FastQ files, a head-crop was performed in order to remove the 12 first bases using the Trimmomatic tool (38). Mapping to the S.scrofa 11.1 reference genome was performed using the short read gapped-mapper STAR (39). Read quantification for each gene was performed with HTSeq (40) using strict intersection mode and a minimum alignment quality of 10. After filtering out low abundant genes (mean counts <10), DESeq2 (41) with outlier replacement and independent filtering was used on the counts matrix to calculate differential abundance. To remove a hidden technical batch effect, Surrogate Variable Analysis (SVA) (42) was used to estimate a batch variable that was added to the DESeq2 formula.

### Bioinformatic Analysis

The STRING preranked enrichment analysis (43) was used to functionally characterize proteome abundance alterations between genotypes (MIDY vs. WT) and tissue types (SCAT vs. MAT). Signed log-transformed *P*-values were used as ranking metric and FDR stringency was set to 0.01. To reduce redundancy, significant Gene Ontology (GO) biological processes were grouped into similar ontological terms with REVIGO (44) at an allowed similarity of 0.5 for the genotype comparison and 0.4 for the tissue type comparison, respectively.

To integrate proteomics and RNA-seq data, protein abundance ratios and DESeq2-normalized mRNA abundance ratios for common identifications were combined and subjected to a statistically controlled 2D annotation enrichment analysis (45). Protein and RNA-seq abundance ratios were separately rank-transformed and are shown as MIDY/WT proteome and transcriptome score, respectively. Statistical enrichment was determined by a two-dimensional generalization of the non-parametric two-sample test. False discovery rate was controlled by correcting for multiple hypothesis testing. The significant cutoff for correlating, non-correlating and anti-correlating GO and KEGG annotations was set to FDR <0.1.

### Leptin RIA

Serum leptin levels were measured using a multi-species leptin radioimmunoassay (Cat. # XL-85K; EMD Millipore Corporation) that has been validated for porcine samples (46). Data were transformed to natural logarithms to approximate normal distribution and analyzed by student's *t*-test.

### Histology and Quantitative Morphological Analyses

For histological and quantitative morphological analyses of adipocytes in WT and MIDY pigs, tissue samples were collected from the SCAT and MAT adipose tissue depots, as described previously (29, 30, 47). Isotropic uniform random (IUR) cryo-sections (30, 47, 48) of 10 μm nominal section thickness were prepared and stained with the Periodic acid–Schiff (PAS) reaction. Quantitative morphological analyses were performed, using an automated stereology system with NewCast software (Visiopharm, Denmark). In systematically randomly sampled fields of view, adipocyte cross section profiles were sampled with point-sampled-intercepts and unbiased counting frames at 10x objective magnification (48, 49). The volume weighted mean adipocyte volumes were determined, using the nucleator method (50–52). Per case, 107 ± 6 measurements (mean ± SD) were taken, on the average. For demonstration of the adipocyte histomorphology, additional hematoxylin and eosin-stained sections of paraffin-embedded adipose tissue samples were prepared. The volume weighted mean adipocyte volumes in SCAT and MAT adipose tissue depots were statistically analyzed, using GraphPad PRISM (version 9.1.1., GraphPad Software, USA). Data are presented as means and single values and standard deviations. Data distributions were analyzed, using Shapiro-Wilk tests. Mean adipocyte volumes in SCAT and MAT adipose tissue depots of the same animals were compared, using paired student *t*-tests (normally distributed data). Mean SCAT- and MAT-adipocyte volumes of WT vs. MIDY pigs were compared by student *t*-tests (normally distributed data). *P*-values <0.05 were considered significant.

## Results

### Mass Spectrometry-Based Proteome Analysis of Adipose Tissue Depots From Insulin-Deficient Diabetic Pigs

To explore the molecular effects of chronic, absolute insulin deficiency and hyperglycemia on AT depots, we performed a label-free liquid chromatography-tandem mass spectrometry analysis (LC-MS/MS) of MAT and SCAT samples from MIDY (*n* = 4) and WT (*n* = 5) animals.

**Figure 1:**
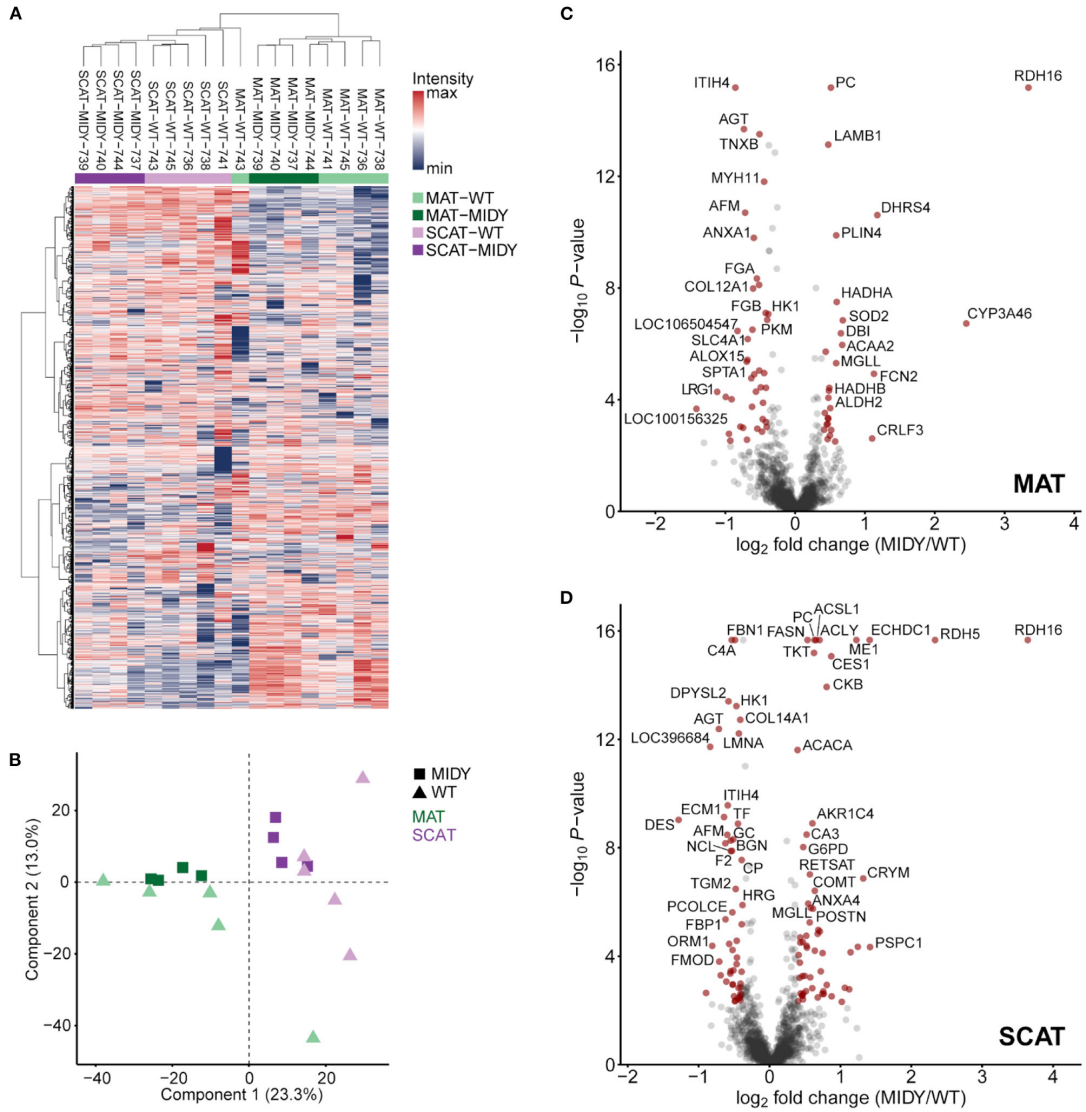
Quantitative proteome analysis of mesenteric (MAT) and subcutaneous (SCAT) adipose tissue from MIDY and WT pigs. **(A)** Unsupervised hierarchical clustering of normalized protein intensities of MAT and SCAT from MIDY (*n* = 4) and WT (*n* = 5) animals. The color code indicates normalized intensity values. **(B)** Principal component analysis (PCA) indicates clustering of individual sample groups. Spots represent individual animals. Green and purple colors indicate MAT and SCAT samples, and squares and triangles indicate MIDY and WT samples, respectively. **(C,D)** Volcano plots visualize the quantitative proteome alterations in MAT **(C)** and SCAT **(D)** from MIDY vs. WT pigs. Red spots indicate differentially abundant proteins (fold-change > 1.3 and Benjamini-Hochberg-adjusted *P*-value < 0.05).

We identified a total of 23,730 peptides from 2,851 proteins with high confidence (FDR <0.01). A full list of all identified proteins can be found in Supplementary Table 1. Unsupervised hierarchical clustering of normalized protein intensities (Figure 1A) and a principal component analysis (Figure 1B) show a clear separation of tissue types (MAT and SCAT) and indicate clustering of genotypes (MIDY and WT).

### Visceral Mesenteric vs. Subcutaneous Adipose Tissue

We used an MS-EmpiRe workflow (36), to detect quantitative proteome differences between the two adipose tissue types within genotype. In WT pigs, 331 proteins were found to be differentially abundant (fold-change > 1.3 and Benjamini-Hochberg-adjusted *P*-value < 0.05) between SCAT and MAT (Supplementary Figure 1A, Supplementary Table 2). In MIDY pigs, 371 proteins were significantly different in abundance between the fat depots (Supplementary Figure 1B, Supplementary Table 3).

**Figure 2:**
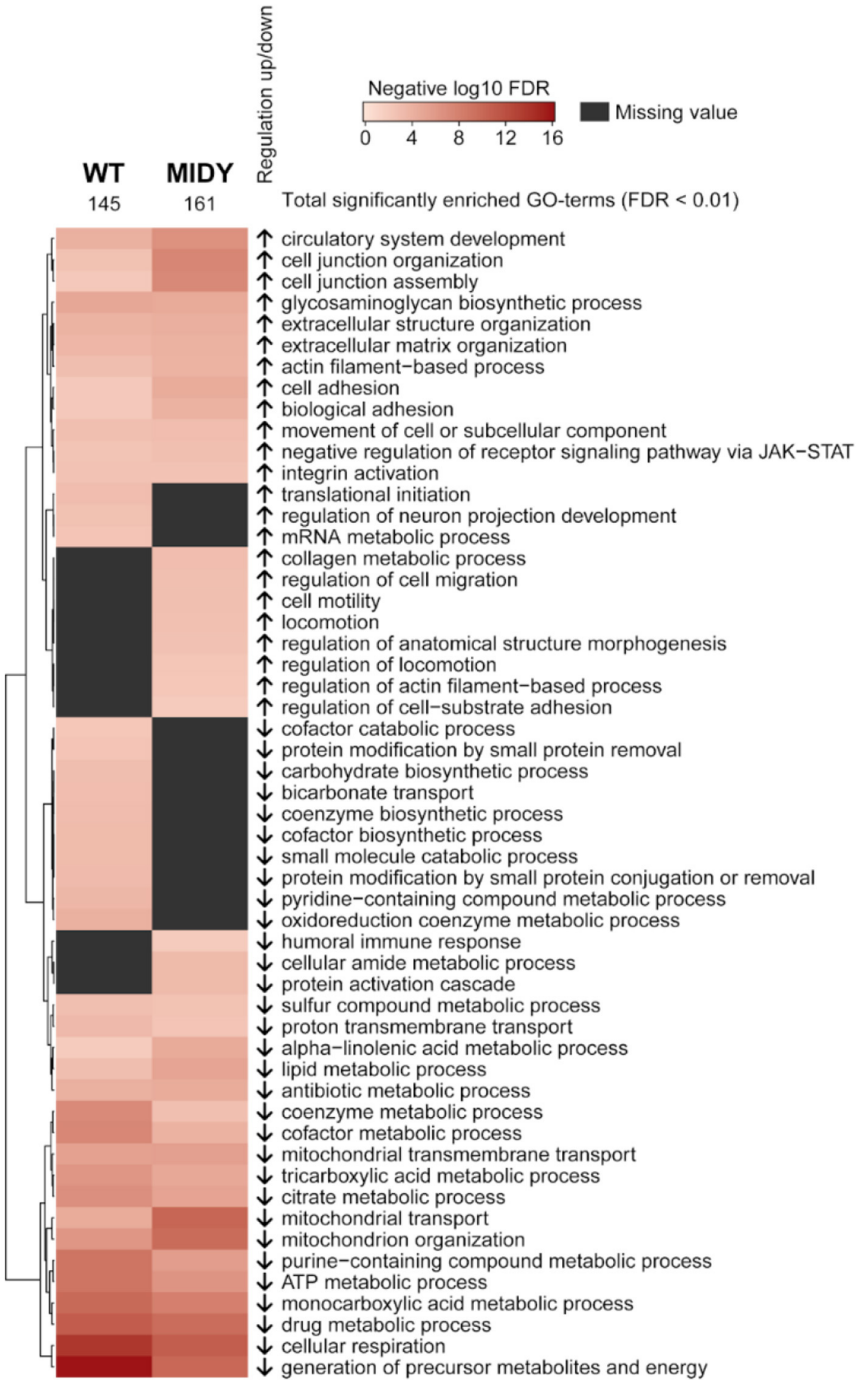
Functional characterization of adipose tissue depot differences between MAT and SCAT in WT and MIDY animals. Heatmap shows GO-term enrichment in SCAT compared to MAT. Enrichment analysis was performed using a pre-ranked STRING analysis and an FDR cutoff of 0.01. Significantly enriched GO biological processes were summarized with REVIGO by grouping semantically similar ontology terms. Arrows indicate regulation in SCAT.

A STRING pre-ranked functional enrichment analysis (43) of proteome profiles from MAT and SCAT was done to reveal tissue-specific signatures for WT and MIDY animals. From the GO biological processes database, 145 and 161 terms were found in WT and MIDY, respectively, to be significantly enriched (FDR < 0.01) in SCAT vs. MAT (Supplementary Tables 4, 5). Similar ontology terms were revealed with REVIGO (44) and the resulting clusters are visualized in Figure 2. SCAT from WT and MIDY animals showed, among others, a distinct enrichment of protein complexes involved in cell junction assembly and organization, of collagens involved in extracellular matrix organization and of actin filaments. On the other hand, proteins related to mitochondrial respiration and to glucose and lipid metabolism were overrepresented in MAT compared to SCAT, the former more pronounced in MIDY and the latter more pronounced in WT. Specifically, proteins involved in pyruvate metabolism, TCA cycle, oxidative phosphorylation (OxPhos), fatty acid biosynthesis and beta oxidation were consistently increased in MAT of both WT and MIDY animals.

### Adipose Tissues From Diabetic vs. Non-diabetic Pigs

The quantitative comparison of both fat depot proteomes from diabetic MIDY and non-diabetic WT animals led to 68 differentially abundant proteins (fold-change > 1.3 and Benjamini-Hochberg-adjusted *P*-value < 0.05) in MAT (Figure 1C, Supplementary Table 6) and 112 proteins in SCAT (Figure 1D, Supplementary Table 7). In both AT depots, retinol dehydrogenase 16 (RDH16) was the protein with the highest abundance increase in MIDY, with log2 fold-changes of 3.3 in MAT and 3.6 in SCAT, respectively. Likewise, further proteins involved in retinol metabolism were increased in abundance, namely apolipoprotein A-IV (APOA4) and aldo-keto reductase family 1 (AKR1C4) in both AT depots, dehydrogenase/reductase 4 (DHRS4) exclusively in MAT and retinol dehydrogenase 5 (RDH5) and retinol saturase (RETSAT) exclusively in SCAT. The group of proteins with the strongest decrease in both MIDY AT depots contained, among others, a large number of serpin family A members (e.g., serpin A3-6, serpin A3-8, and SERPING1) as well as leucine-rich repeat (LRR)-containing proteins, e.g., biglycan (BGN), fibromodulin (FMOD), and leucine rich alpha-2-glycoprotein 1 (LRG1) linked to collagen fibril organization and immune response.

**Figure 3:**
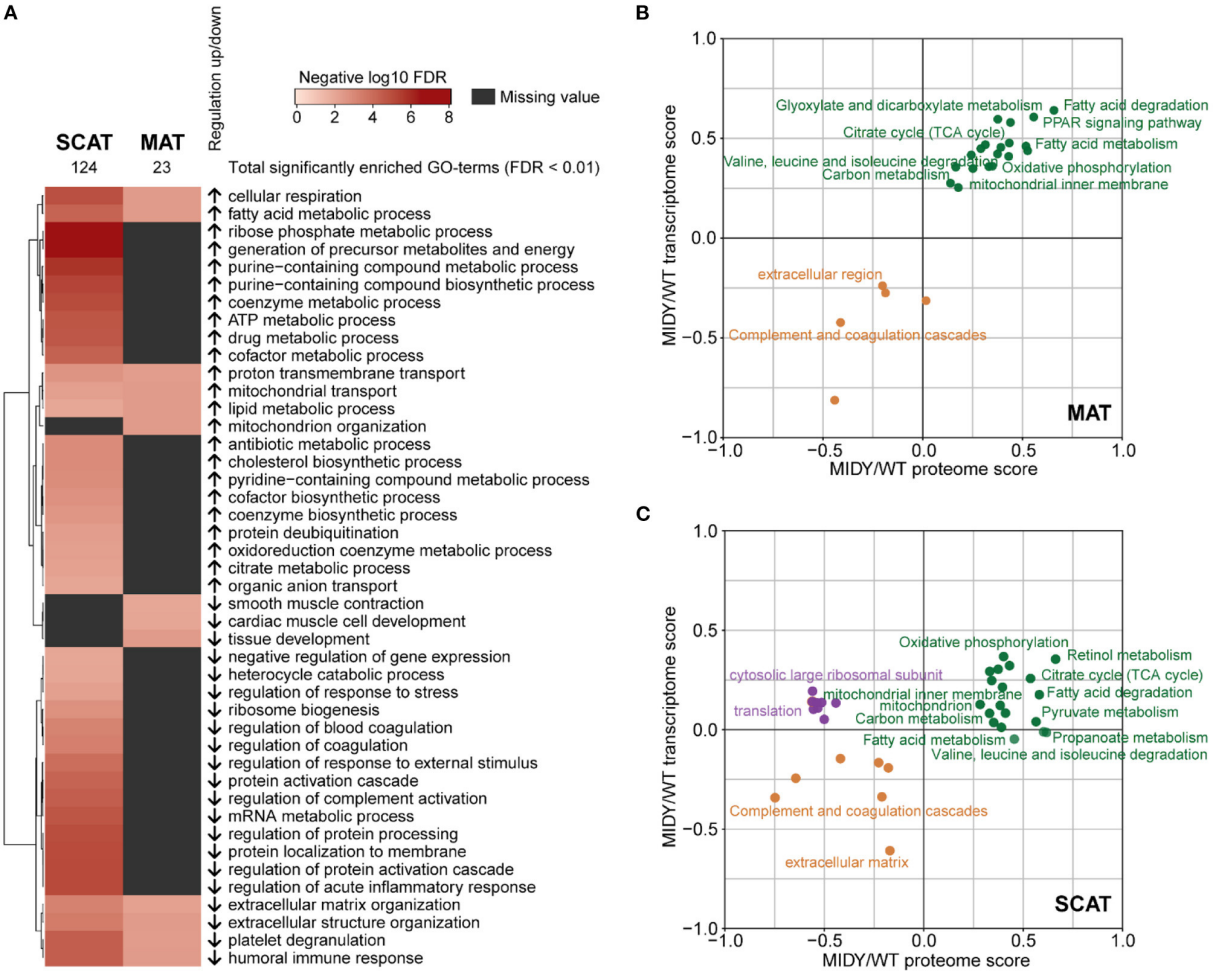
Multi-omic functional characterization of MIDY adipose tissue **(A)** Annotation enrichment analysis of SCAT and MAT proteome alterations in MIDY. Heatmap shows GO-term enrichment in MIDY compared to WT. Enrichment analysis was performed using a pre-ranked STRING analysis and an FDR cutoff of 0.01. Significantly enriched GO biological processes were summarized with REVIGO by grouping semantically similar ontology terms. Arrows indicate regulation in MIDY. **(B,C)** 2D annotation enrichment analysis showing the correlation of proteomics and transcriptomics data. Fold-changes in the proteome (x-axis) and transcriptome (y-axis) are rescaled and shown as scores. Terms depicted in green dots show a common upregulation at the transcriptome and proteome level. Terms in orange show a common downregulation. Purple dots indicate downregulation at the proteome level and upregulation of corresponding mRNA levels. Significant KEGG pathways and Gene Ontology categories with FDR < 0.1 are shown.

To functionally characterize proteome alterations, a STRING pre-ranked annotation enrichment analysis was performed on both AT depot datasets. From the GO biological processes database, 23 terms were significantly enriched (FDR < 0.01) in MAT and 124 in SCAT, respectively (Figure 3A). Proteins more abundant in MIDY vs. WT of MAT and SCAT were enriched for terms related to fatty acid and lipid metabolism as well as to mitochondrial respiration, while proteins involved in extracellular matrix organization, immune response and platelet degranulation are more likely to be reduced in MIDY ATs. Terms related to processes relevant for metabolite and energy production, e.g., citrate and purine metabolism were predominantly enriched in the set of proteins more abundant in MIDY vs. WT SCAT, while proteins with reduced abundance were found to be involved in regulation of coagulation and regulation of protein activation cascades. The detailed results of the STRING analysis are provided in Supplementary Tables 8, 9.

**Figure 4:**
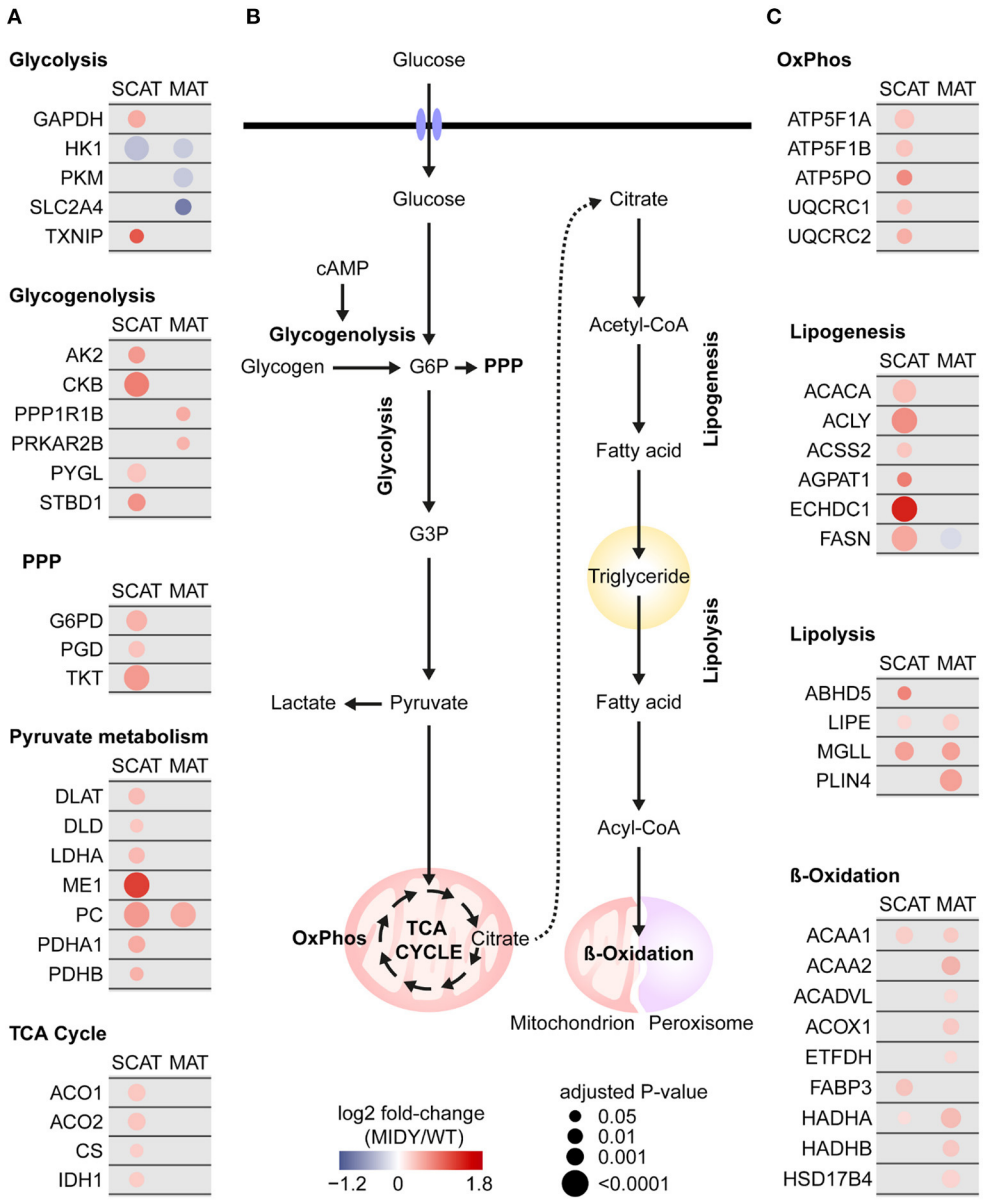
Impaired carbohydrate and fat metabolism in MIDY SCAT and MAT. **(A,C)** Protein abundance alterations in MIDY compared to WT are color coded and shown as log2 fold-change of MIDY/WT. Red circles represent an increased abundance and blue a decreased abundance in MIDY, respectively. Circle size correlates with Benjamini-Hochberg-adjusted *P*-value. Only proteins with an adjusted *P* < 0.05 in at least one tissue depot are shown. Proteins that were not significant or not detected in that tissue depot are grayed out. **(B)** Schematic representation of glucose and fat metabolism in adipose tissue. G6P, glucose-6-phosphate; G3P, glyceraldehyde-3-phosphate; cAMP, cyclic adenosine monophosphate; PPP, pentose phosphate pathway; OxPhos, oxidative phosphorylation.

A strikingly large part of proteins commonly increased in both tissue depots from MIDY pigs are known to be involved in carbohydrate and lipid metabolism, e.g., pyruvate carboxylase (PC), monoglyceride lipase (MGLL) and acetyl-CoA acyltransferase 1 (ACAA1) (Figures 4A–C). Furthermore, well-known metabolic and regulatory enzymes of glucose import were significantly reduced in abundance in MIDY vs. WT pigs, among them hexokinase-1 (HK1) in both depots and solute carrier family 2, facilitated glucose transporter member 4 (SLC2A4/GLUT4) in MAT only. Remarkably, proteins associated with subsequent glucose metabolic pathways (e.g., glycogenolysis, pentose phosphate pathway, pyruvate metabolism, TCA cycle, and mitochondrial oxidative phosphorylation) were consistently more abundant in SCAT from MIDY compared to WT pigs. Although a similar trend was visible for MAT, the changes were less pronounced, and the vast majority was only significant in SCAT. A similar pattern was observed for proteins involved in lipogenesis, the step to provide free fatty acids from acetyl-CoA, where SCAT showed stronger abundance alterations compared to MAT.

### Integrative Analysis of Transcriptomics and Proteomics Data

To investigate transcriptional regulation of insulin deficiency and hyperglycemia on AT, we additionally performed RNA-sequencing of the same tissue specimens from MIDY (*n* = 4) and WT (*n* = 5) animals as used for proteomics experiments (Supplementary Tables 10, 11). To detect correlated and uncorrelated functional changes, we performed a 2D annotation enrichment analysis of the transcriptome and proteome data (Figures 3B,C) (45). We observed a highly concordant enrichment (FDR < 0.1) of processes related to e.g., fatty acid metabolism, TCA cycle and oxidative phosphorylation in MAT and SCAT of MIDY pigs, and a concordant decrease of terms related to extracellular matrix, the complement system and coagulation cascades in both datasets. Notably, in SCAT from MIDY pigs, we detected a strong enrichment of genes involved in retinol metabolism on both the mRNA and protein level. A discordant pattern could be observed in SCAT, where proteins linked to translational regulation as well as cytosolic large ribosomal subunits showed a decrease in MIDY, while RNA expression levels were slightly elevated in MIDY.

### Histology, Quantitative Morphological Analyses and Leptin Measurements

SCAT and MST adipose tissue samples of WT and MIDY pigs displayed a regular histomorphology without evidence of histopathological alterations. In both WT and MIDY pigs, the (volume weighted) mean volumes of adipocytes in the MAT were significantly larger than in the SCAT, whereas the adipocyte volumes of corresponding adipose tissue depots did not significantly differ in WT vs. MIDY pigs (Supplementary Figures 2A,B). To address leptin signaling, we measured serum leptin concentrations and found significantly reduced levels (*p* = 0.03) in MIDY pigs (Supplementary Figure 3).

## Discussion

Adipose tissue plays a central role in energy homeostasis and metabolic function [reviewed in (2, 53)], and its development and functions are regulated by insulin signaling (54). Metabolic and functional characteristics differ between adipose tissue depots and their specific contribution to metabolic health and disease is extensively studied (9, 14, 55–57).

Proteomics provides holistic insights into adipose tissue functions in health and disease. A number of studies investigated proteome profiles of adipose tissue in type 2 diabetes mellitus (18–22), which is characterized by insulin resistance and hyperinsulinemia. However, holistic studies of adipose tissue in insulin-deficient diabetes mellitus are lacking.

Therefore, we analyzed fat tissue samples from long-term diabetic (2 years) *INS*^C94Y^ transgenic pigs, a model for mutant *INS* gene induced diabetes of youth (MIDY) (23), and wildtype (WT) littermates. The samples were taken according to the principles of random systematic sampling and archived in the Munich MIDY pig biobank (29). To explore how chronic, absolute insulin deficiency and hyperglycemia affect transcriptomes and proteomes of different adipose tissue types, a multi-omics analysis of MAT and SCAT from MIDY and WT animals was performed.

### Proteome Signatures of Subcutaneous and Visceral Adipose Tissue

Adipocytes in different adipose tissue depots have different morphology and fulfill different tasks. They also differ in their reaction toward obesity (adaptive growth patterns)—see Stenkula and Erlanson-Albertsson (58) and recent quantitative morphological analyses of adipose tissue in porcine biomedical models by Theobalt et al. (59). In line with these findings we found major histological differences between SCAT vs. MAT tissue samples of MIDY and WT pigs. Moreover, using a functional enrichment analysis of protein profiles from MAT and SCAT depots from WT and MIDY pigs, we detected distinct proteomic patterns that reflect depot-specific functions. Among the most enriched gene sets in SCAT vs. MAT were clusters related to extracellular matrix (ECM) organization, including significantly higher abundances of multiple subunits of type I, IV, VI, XIV collagens, fibronectin, as well as B2 and A5 subunits of laminin. This agrees with previous DNA microarray and histological observations, suggesting that the ECM of SCAT maintains a fibrous network connecting dermis and subdermal tissues (60). Similarly, a depot-specific heterogeneity of adipose tissue in ECM composition was reported in previous studies, suggesting ECM as determining factor for adipogenic capacity and a greater adipogenic potential for subcutaneous fat (61, 62). Furthermore, we detected higher abundances of proteins related to cell junction assembly, cell adhesion, and actin cytoskeleton organization in SCAT compared to MAT, which additionally highlights the structural function of SCAT.

The proteome of MAT revealed an enrichment of proteins related to metabolic processes. In particular, glucose and lipid metabolic pathways, including pyruvate metabolism, fatty acid synthesis and degradation were found to be overrepresented. Pertinent metabolic pathways, such as tricarboxylic acid (TCA) cycle, oxidative phosphorylation, and fatty acid oxidation, are localized in the mitochondria (63). In rats and humans, it was shown that mitochondrial content is higher in visceral compared with subcutaneous adipose tissue (64, 65). Accordingly, there was a significantly higher abundance of proteins representing these pathways in MAT. Taken together, our data support the notion that MAT is metabolically more active and more sensitive to mitochondrial substrate supply than SCAT.

### Proteome Alterations Between MIDY and WT Reflects Adipose Depot-Specific Response to Insulin Deficiency and Hyperglycemia

The comparison of SCAT and MAT from MIDY pigs with age-matched WT controls revealed significant depot-dependent transcriptome and proteome alterations related to glucose and lipid homeostasis in both AT depots of MIDY pigs. Insulin signaling is fundamental for the regulation of energy and lipid metabolism in adipose tissue [reviewed in (66, 67)]. It promotes glucose uptake into adipocytes by coordinating the translocation of the glucose transporter type 4 (SLC2A4 alias GLUT4) from intracellular sites to the cell surface (68). Impaired GLUT4 translocation is an early sign of developing insulin resistance and type 2 diabetes mellitus (69).

In MAT from MIDY pigs, the abundance of GLUT4 was significantly decreased compared to WT pigs, indicating that not only the membrane translocation but also the absolute expression level of GLUT4 is insulin dependent. Interestingly, GLUT4 was not significantly altered in SCAT from MIDY pigs, suggesting depot-specific regulatory mechanisms of its abundance. Indeed, it was reported that, compared with SCAT, visceral AT has increased insulin-stimulated glucose uptake (70–72) and that insulin signaling was more pronounced in visceral AT than in SCAT (73, 74). This suggests that in MIDY pigs, insulin insufficiency has a stronger impact on GLUT4-mediated glucose uptake in MAT than in SCAT.

The thioredoxin-interacting protein (TXNIP) was strongly increased in abundance in the SCAT samples from MIDY pigs. *TXNIP* transcription is induced by glucose and concurrently, the TXNIP protein suppresses excess cellular glucose uptake. It is therefore described as central regulatory element for acute energy stress response (75). In MIDY pigs, the upregulation of TXNIP can therefore be interpreted as a response of adipose tissue to permanently elevated glucose levels.

After entering the cell, the initial step in glucose metabolism is phosphorylation, catalyzed by hexokinases. Strikingly, hexokinase 1 (HK1), a proposed key regulator of AT glucose uptake (70), was found to be significantly decreased in both MIDY AT depots. Overall, our data suggests an impaired glucose import and a reduced glucose phosphorylation in MIDY MAT and SCAT cells.

The HK1 reaction product glucose-6-phosphate (G6P) can be metabolized in several alternative pathways, namely downstream glycolysis, pentose phosphate pathway (PPP) and glycogen metabolism (76). Surprisingly, despite the presumed reduced glucose import and hexokinase-catalyzed phosphorylation in MIDY AT depots, we detected a consistently higher abundance of key enzymes acting in subsequent glycolytic steps as well as in PPP in MIDY SCAT. This inverse correlation of reduced glucose uptake and phosphorylation to G6P with a simultaneous enhanced glycolytic degradation of G6P was previously described as “hexokinase paradox” (77). A possible cause for a boosted glycolytic degradation could be excess glycogenolysis, which demands glycolytic enzymes for metabolizing glycogen-derived G6P. The rate of glycogen breakdown is critically insulin-dependent, and it was shown that during insulin deficiency, glycogenolysis is increased (78). Indeed, we found elevated levels of glycogen phosphorylase (PYGL), the rate-limiting enzyme in glycogenolysis, as well as of multiple enzymes involved in the activation of glycogenolysis such as creatine kinase B-type (CKB) and adenylate kinase 2 (AK2), advocating a stimulated glycogenolysis in MIDY SCAT.

Glycolysis leads to the generation of pyruvate, which can enter the TCA cycle in mitochondria or can be reduced to lactate by lactate dehydrogenase (79). The abundance of lactate dehydrogenase (LDHA) was significantly increased in MIDY SCAT, and did not change in MIDY MAT compared to WT. Interestingly, Markan et al. also observed an increased lactate production in epididymal adipocytes with elevated glycogenolysis, suggesting a concurrent regulation (80). Since excess lactate can cause intracellular acidosis, it can be released to preserve continuity of glycolysis. An important compensatory mechanism to regulate intracellular pH is the import of HCO${}_{3}^{-}$, which can be interconverted to CO_2_, through the SLC4 family of transporters. This reversible reaction is catalyzed by carbonic anhydrases [reviewed in (81)]. Strikingly, both the bicarbonate transporter SLC4A1 (alias AE1) as well as multiple carbonic anhydrases (CA1, CA3, CA5B, CA2 as a trend) were detected at increased levels in MIDY SCAT compared to WT, suggesting an active regulation of pH in MIDY SCAT. An alternative use for lactate was proposed recently, namely that it can serve as a rich carbon source and fuel mitochondrial TCA cycle in normal and tumor tissue (82).

Inside mitochondria, the pyruvate dehydrogenase complex (PDC) converts pyruvate into acetyl-CoA to enter TCA cycle for energy production. The consistent increase of PDC enzymes including pyruvate dehydrogenase E1 subunits alpha and beta (PDHA1 and PDHB), dihydrolipoamide S-acetyltransferase (DLAT) and dihydrolipoamide dehydrogenase (DLD), as well as higher levels of citrate synthase (CS), the rate-limiting enzyme of the TCA cycle, which catalyzes the condensation of acetyl-CoA with oxaloacetate, indicate an enhanced TCA cycle flux in MIDY SCAT compared to WT. As an alternative, pyruvate can be transformed to oxaloacetate by pyruvate carboxylase (PC) to replenish the TCA cycle intermediates (83). Accordingly, PC levels were found to be increased in both AT depots, suggesting an enhanced oxaloacetate generation to cover an increased demand for pathway components.

Adipocytes serve primarily as energy storage for excess nutrients and on the other hand regulate lipid mobilization and distribution in the body. During *de novo* lipogenesis (DNL), excess carbohydrates are converted into fatty acids (FA), which can be stored as triacylglycerides within lipid droplets [reviewed in (1, 84, 85)]. The main substrate for *de novo* synthesis of fatty acids is acetyl-CoA, which can either be generated from citrate by ATP-citrate lyase (ACLY) or from acetate catalyzed by acetyl-CoA synthetase 2 (ACSS2). In the first and rate-limiting step of DNL, acetyl-CoA is transformed into malonyl-CoA by acetyl-CoA carboxylases (ACCs). Malonyl-CoA undergoes a condensation reaction with acetyl-CoA by fatty acid synthase (FASN) in the presence of PPP-produced NADPH to generate triglycerides. In SCAT of MIDY pigs, we found elevated levels of ACLY and ACSS2, which provide metabolic substrates for lipogenesis. Consequently, we observed an increased abundance of G6PD, the rate-limiting PPP enzyme, concomitant with elevated levels of key lipogenic enzymes, such as ACACA/ACC1 and FASN. In contrast, FASN abundance was reduced in MAT of MIDY pigs, suggesting a limited FASN-driven fatty acid synthesis in this fat depot. This might be associated with the significantly reduced levels of the GLUT4/SLC2A4 glucose transporter in MAT of MIDY pigs, as it was shown that adipose tissue lipogenesis strongly correlates with insulin sensitivity (86) and that GLUT4 overexpression in mice led to an elevated AT lipogenesis (87). To compensate the lack of fatty acids from endogenous lipogenesis, MAT might obtain fatty acids from exogenous uptake by passive diffusion or specialized transporters (88–90). Using this route, fatty acids released from lipolysis might be reimported *via* the so-called fatty acid recycling pathway (91). Alternatively, consumption of lipid droplet reserves could help to secure the endogenous FA pool.

Triglyceride turnover is crucial for lipid homeostasis in adipose tissue. Breakdown of triglycerides *via* lipolysis enables release of glycerol and non-esterified fatty acids (NEFAs) which can serve as energy substrates in mitochondrial β-oxidation or can be released and fuel energy metabolism in other organs (92, 93). It is known that insulin suppresses lipolysis and promotes triglyceride storage in adipocytes by diminishing expression of lipolysis-specific enzymes (94–96).

Consequently, in MIDY pigs, we observed an increase of key enzymes involved in lipolysis in both AT depots, among them hormone-sensitive lipase (LIPE alias HSL) and monoglycerol lipase (MGLL alias MGL), which catalyze the stepwise breakdown of triglycerides to glycerol and FAs (92, 97). In this context, it is worth mentioning that enzymes involved in retinol metabolism were increased in both AT depots of MIDY pigs. In particular, retinol dehydrogenase 16 (RDH16), whose expression is negatively regulated by insulin and which catalyzes the first of the two-step reaction from retinol to retinoic acid (98), was among the most significantly increased proteins in MIDY AT. Remarkably, in the liver of MIDY pigs, together with elevated levels of retinal and retinoic acid, RDH16 was also found to be increased in abundance and was suggested as a key driver of stimulated hepatic gluconeogenesis in MIDY pigs (27). Retinoid action has tissue-specific differences and in AT, elevated retinoic acid was shown to suppress adipogenesis (99) and promote lipolysis (100, 101). It is therefore conceivable that the increase in retinol metabolism promotes lipolysis in MIDY AT. Together, in both depots, fat break-down and release potentially predominate accumulation, which is supported by the absence of adipocyte enlargement and by elevated levels of circulating free fatty acids in MIDY pigs (27). β-oxidation is the central pathway for the degradation of long-chain fatty acids and is often discussed in the context of the pathophysiology of insulin resistance, diabetes, and obesity [reviewed in (102)]. While β-oxidation mainly occurs in mitochondria, peroxisomes are indispensable for metabolizing very-long-chain fatty acids and branched-chain fatty acids (103). Acetyl-CoA produced through oxidative degradation of FAs can fuel the TCA cycle and oxidative phosphorylation to push energy production. Our proteomics data showed that a variety of enzymes, transporter and facilitating proteins involved in fatty acid oxidation were increased in MAT and to a lesser extent in SCAT of MIDY pigs. Increased mRNA levels of the two acyltransferases, carnitine O-palmitoyltransferases 1 and 2 (CPT1A and CPT2), and carnitine acyl carnitine translocase (SLC25A20), the key transporters for fatty acid import into mitochondria (102), point toward an increased FA uptake *via* the carnitine cycle in MAT from MIDY pigs. In the β-oxidation cycle, we found increased levels of major enzymes involved in the stepwise shortening of acyl-CoA in MAT of MIDY pigs, namely the very long chain acyl-CoA dehydrogenase (ACADVL) and the medium-chain 3-ketoacyl-CoA thiolase (ACAA2), as well as the mitochondrial trifunctional subunits alpha (HADHA) and beta (HADHB). Taken together, our data indicates that MAT in MIDY pigs has increased capacities for mitochondrial uptake and oxidative degradation of FAs. Following the concept of the Randle cycle (104), the enhanced β-oxidation in MAT of MIDY pigs might therefore be an adaptation to the reduced availability of glucose, with a metabolic switch from glycolysis to fatty acid oxidation. A potential key mediator thereby might be leptin, as it was reported recently that hypoleptinemia promotes a shift from carbohydrate to fat metabolism *via* the hypothalamic-pituitary-adrenal axis, leading to increased AT lipolysis and hepatic ketogenesis, which is necessary to maintain glucose homeostasis and substrate supply during starvation (105). This hypothesis is strongly supported by a reduction of leptin mRNA in MAT and a significant reduction of circulating leptin levels in MIDY pigs.

Collectively, our multi-omics analysis of MAT and SCAT of MIDY pigs revealed severe depot-specific dysregulations in response to insulin deficiency. Our data indicates regionally different metabolic adaptations to overcome energy stress caused by reduced glucose utilization in MIDY adipocytes. This study provides novel pathophysiologic insights and is an important resource for understanding adipocyte functions in insulin-deficient diabetes.

## Data Availability Statement

The datasets presented in this study can be found in online repositories. The mass spectrometry proteomics data generated and analyzed during the current study have been deposited to the ProteomeXchange Consortium via the PRIDE (106) partner repository, http://proteomecentral.proteomexchange.org; PXD026910. The transcriptomics results are included in the article/Supplementary Material.

## Ethics Statement

All experiments were performed according to the German Animal Welfare Act with permission from the responsible authority (Government of Upper Bavaria), following the ARRIVE guidelines and Directive 2010/63/EU for animal experiments.

## Author Contributions

FF, EL, BS, and TF: proteomics studies and data analysis. MB, JP-M, and HB: transcriptomics studies and statistics. AB, SR, RW, and EW: generation of animal model and providing samples. MH and GA: investigation. FF, EL, BS, TF, and EW: conceptualization and writing. All authors read and approved the final manuscript prior to submission.

## Funding

This study was supported by the German Center for Diabetes Research (DZD e.V.) and the German Research Council (Graduate School QBM; TRR127). This project has received funding from the European Union's Horizon 2020 research and innovation programme under the Marie Skłodowska-Curie Grant Agreement No. 812660 (DohART-NET).

## Conflict of Interest

The authors declare that the research was conducted in the absence of any commercial or financial relationships that could be construed as a potential conflict of interest.

## Publisher's Note

All claims expressed in this article are solely those of the authors and do not necessarily represent those of their affiliated organizations, or those of the publisher, the editors and the reviewers. Any product that may be evaluated in this article, or claim that may be made by its manufacturer, is not guaranteed or endorsed by the publisher.

## Supplementary Material

Supplementary Figure 1 Volcano plots visualize the quantitative proteome differences of SCAT vs. MAT in WT **(A)** and MIDY **(B)** animals. Red spots indicate differentially abundant proteins (fold-change > 1.3 and Benjamini-Hochberg-adjusted *P*-value < 0.05).

Supplementary Figure 2 **(A)** Histology of MAT and SCAT adipose tissue depots in WT and MIDY animals. Paraffin sections, hematoxylin and eosin staining. Bar = 100 μm. **(B)** Volume weighted mean adipocyte volume in MAT and SCAT adipose tissue depots in WT and MIDY animals. Data are means and standard deviations. Data points corresponding to individual animals are indicated by individual symbols. The mean adipocyte volumes in SCAT and MAT adipose tissue depots of the same animals were compared, using paired student *t*-tests. ^*^*p* < 0.05; ^**^*p* < 0.01. Mean SCAT- and MAT-adipocyte volumes of WT vs. MIDY pigs were compared by student *t*-tests, as indicated. *n.s*., not significant (*p* > 0.05).

Supplementary Figure 3 Absolute quantification of leptin levels in sera from WT and MIDY pigs. The difference between the groups was evaluated using a student *t*-test.

Supplementary Table 1 Proteins Identified and quantified by nano-LC-MS/MS-based proteomics.

Supplementary Table 2 Results of MS-EmpiRe-based quantitative proteomics of SCAT vs. MAT from WT animals. Proteins showing significant differences in abundance between MIDY and WT animals (FDR < 0.05, fold-change > 1.3) are marked with a “+.” Positive log2 fold changes means more abundant in the SCAT group.

Supplementary Table 3 Results of MS-EmpiRe-based quantitative proteomics of SCAT vs. MAT from MIDY animals. Proteins showing significant differences in abundance between MIDY and WT animals (FDR < 0.05, fold-change > 1.3) are marked with a “+.” Positive log2 fold changes means more abundant in the SCAT group.

Supplementary Table 4 STRING functional enrichment analysis from SCAT vs. MAT from WT animals. Direction “bottom” indicates enrichment in SCAT compared to MAT, “top” indicates enrichment in MAT, and “both ends” indicates enrichment on both sides of the ranked quantification data. Gene Ontology biological processes with FDR < 0.01 are listed.

Supplementary Table 5 STRING functional enrichment analysis from SCAT vs. MAT from MIDY animals. Direction “bottom” indicates enrichment in SCAT compared to MAT, “top” indicates enrichment in MAT, and “both ends” indicates enrichment on both sides of the ranked quantification data. Gene Ontology biological processes with FDR < 0.01 are listed.

Supplementary Table 6 Results of MS-EmpiRe-based quantitative proteomics of MIDY vs. WT pig visceral mesenteric adipose tissue. Proteins showing significant differences in abundance between tissue depots of MIDY and WT animals (FDR < 0.05, fold-change > 1.3) are marked with a “+.” Positive log2 fold changes means more abundant in the MIDY group.

Supplementary Table 7 Results of MS-EmpiRe-based quantitative proteomics of MIDY vs. WT pig subcutaneous adipose tissue. Proteins showing significant differences in abundance between tissue depots of MIDY and WT animals (FDR < 0.05, fold-change > 1.3) are marked with a “+.” Positive log2 fold changes means more abundant in the MIDY group.

Supplementary Table 8 STRING functional enrichment analysis from MIDY vs. WT MAT. Direction “bottom” indicates enrichment in MIDY compared to WT, “top” indicates enrichment in WT, respectively. Gene Ontology biological processes with FDR < 0.01 are listed.

Supplementary Table 9 STRING functional enrichment analysis from MIDY vs. WT SCAT. Direction “bottom” indicates enrichment in MIDY compared to WT, “top” indicates enrichment in WT, respectively. Gene Ontology biological processes with FDR < 0.01 are listed.

Supplementary Table 10 Transcriptome analysis of MIDY vs. WT pig visceral mesenteric adipose tissue.

Supplementary Table 11 Transcriptome analysis of MIDY vs. WT pig subcutaneous adipose tissue.
