# Supplementary figures and images for: Differential Effects of Insulin-Deficient Diabetes Mellitus on Visceral vs. Subcutaneous Adipose Tissue—Multi-omics Insights From the Munich MIDY Pig Model
Source: Front Med (Lausanne). 2021 Nov 23;8:751277. doi: 10.3389/fmed.2021.751277 (PMC8650062; doi:10.3389/fmed.2021.751277)

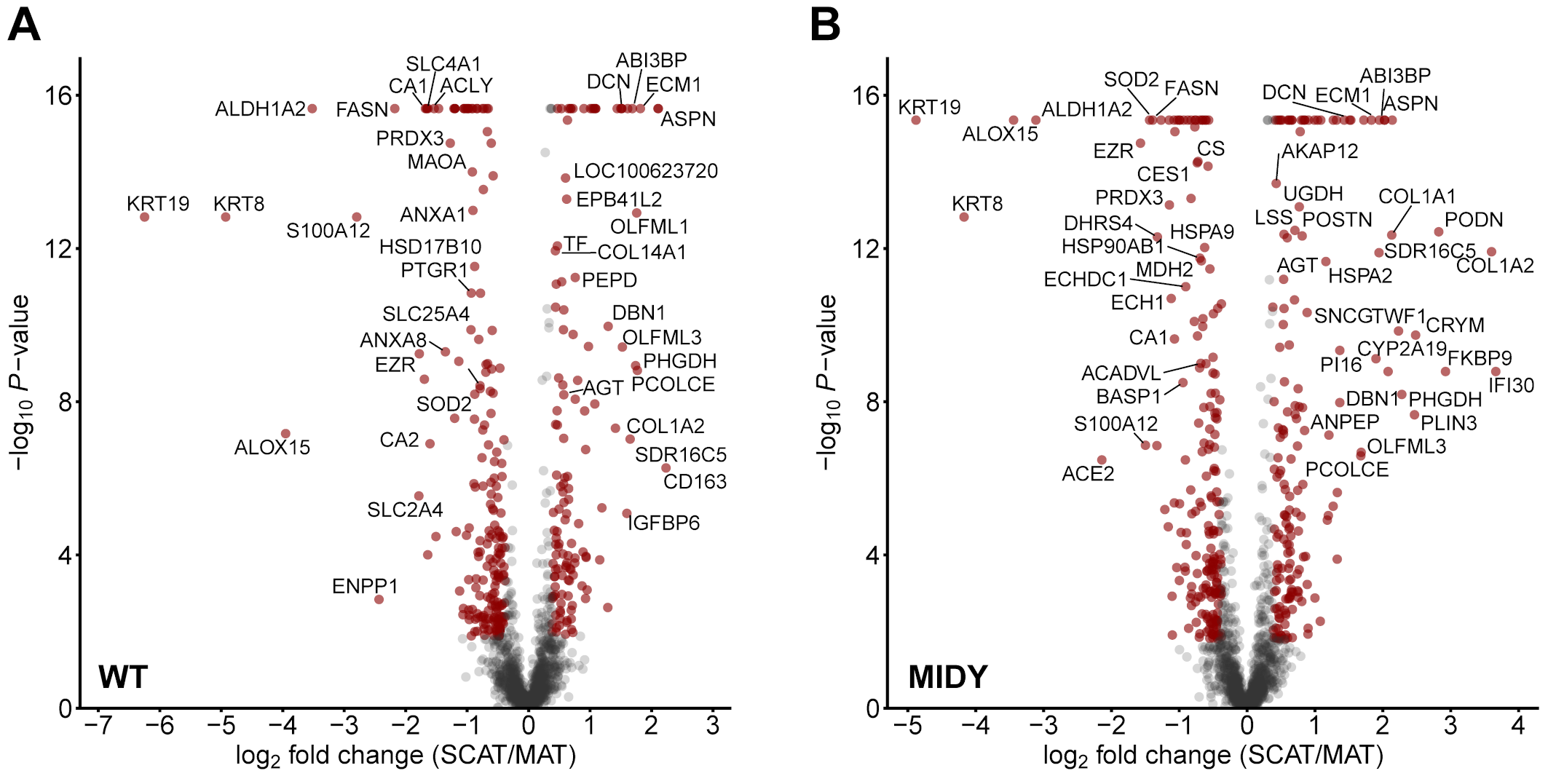

Supplement: Supplementary Figure 1 — Volcano plots visualize the quantitative proteome differences of SCAT vs. MAT in WT (A) and MIDY (B) animals. Red spots indicate differentially abundant proteins (fold-change > 1.3 and Benjamini-Hochberg-adjusted P-value < 0.05). [file Image_1.TIF]

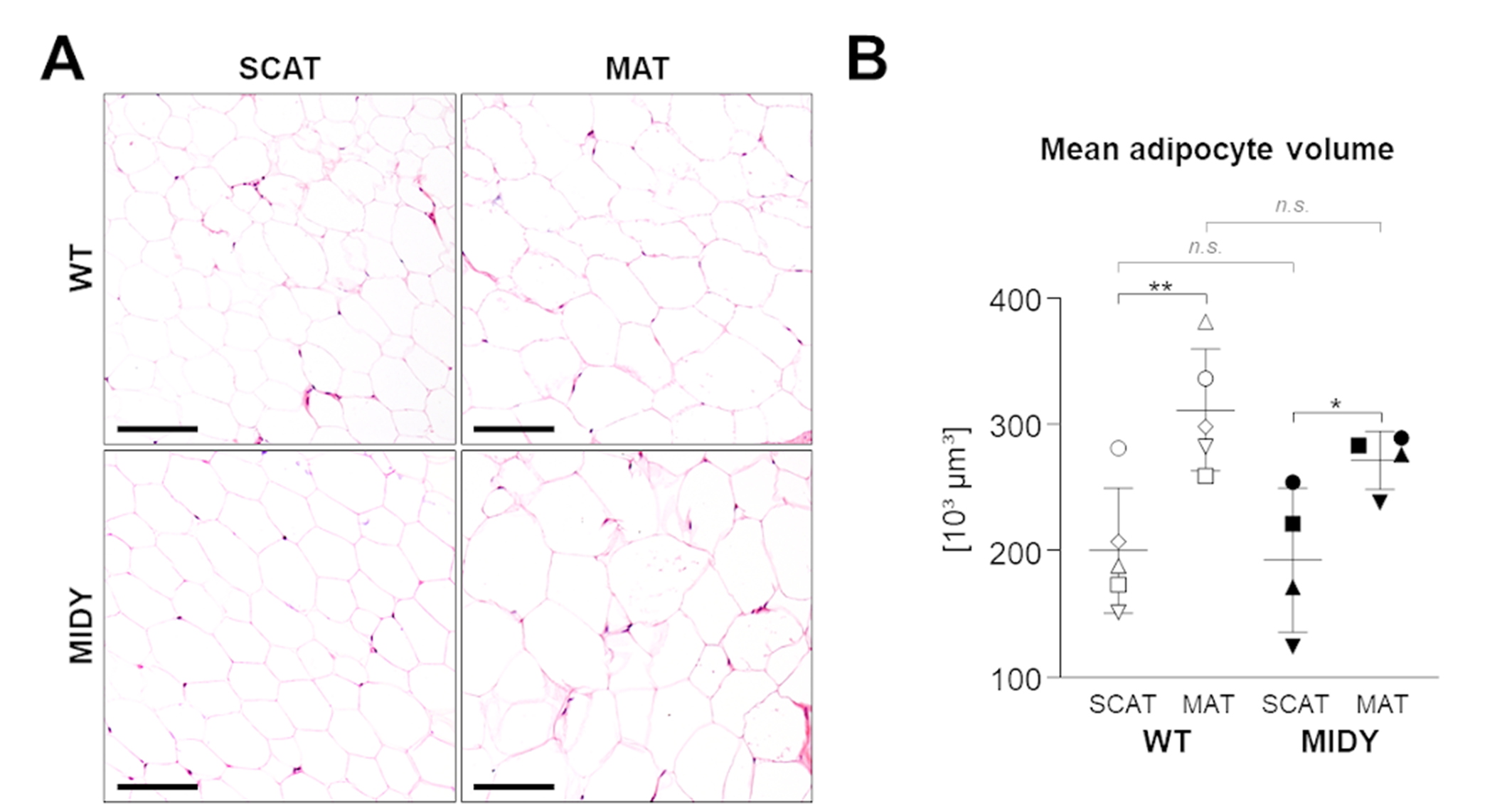

Supplement: Supplementary Figure 2 — (A) Histology of MAT and SCAT adipose tissue depots in WT and MIDY animals. Paraffin sections, hematoxylin and eosin staining. Bar = 100 μm. (B) Volume weighted mean adipocyte volume in MAT and SCAT adipose tissue depots in WT and MIDY animals. Data are means and standard deviations. Data points corresponding to individual animals are indicated by individual symbols. The mean adipocyte volumes in SCAT and MAT adipose tissue depots of the same animals were compared, using paired student t-tests. *p < 0.05; **p < 0.01. Mean SCAT- and MAT-adipocyte volumes of WT vs. MIDY pigs were compared by student t-tests, as indicated. n.s., not significant (p > 0.05). [file Image_2.TIF]

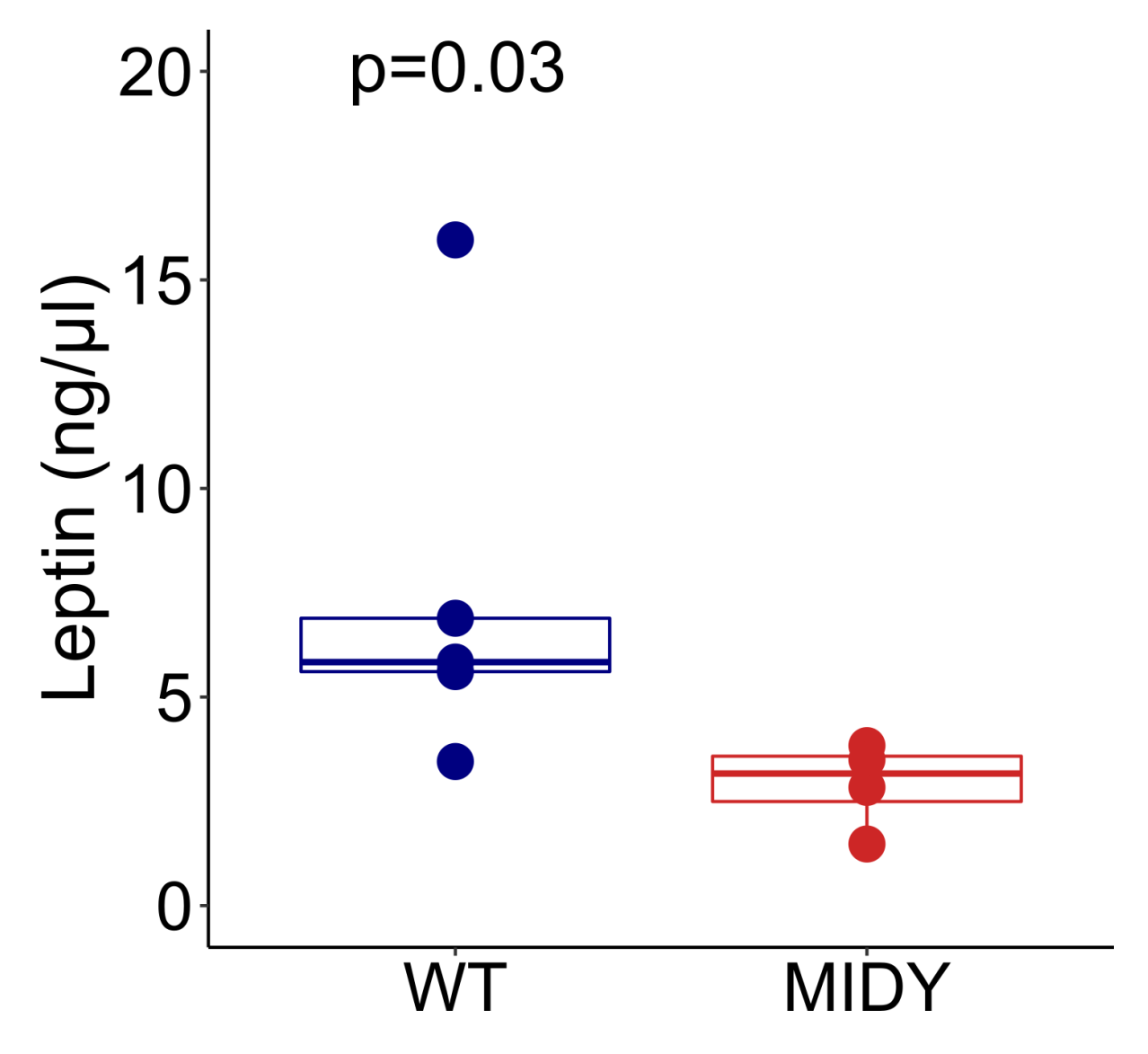

Supplement: Supplementary Figure 3 — Absolute quantification of leptin levels in sera from WT and MIDY pigs. The difference between the groups was evaluated using a student t-test. [file Image_3.TIF]
